# Chikungunya-attributable deaths: A neglected outcome of a neglected disease

**DOI:** 10.1371/journal.pntd.0007575

**Published:** 2019-09-12

**Authors:** Antonio S. Lima Neto, Geziel S. Sousa, Osmar J. Nascimento, Marcia C. Castro

**Affiliations:** 1 Health Surveillance Department, Fortaleza Municipal Health Secretariat (SMS-Fortaleza), Fortaleza, Ceará, Brazil; 2 Health Sciences Center, University of Fortaleza (UNIFOR), Fortaleza, Ceará, Brazil; 3 Takemi Program, Department of Global Health and Population, Harvard T.H. Chan School of Public Health, Boston, Massachusetts, United States of America; 4 Department of Global Health and Population, Harvard T.H. Chan School of Public Health, Boston, Massachusetts, United States of America; DoD - AFHSB, UNITED STATES

Chikungunya is caused by an arbovirus RNA of the genus alphavirus (CHIKV) of Togaviridae family [1]. Symptomatic acute CHIKV infection is mainly characterized by high fever and severe joint pain (arthralgia) that can compromise daily life activities [2]. Acute symptoms (such as fever, myalgia, and exanthema) usually resolve or decrease in intensity (arthralgias) in 1 or 2 weeks, although the acute phase may last up to 21 days [1, 3]. Clinical features of post-acute phase, which begins after 3 weeks of onset of symptoms and may extend for 90 days, include polyarthralgia, polyarthritis, exacerbation of comorbidities, chronic fatigue, and worsening of preexisting degenerative or traumatic arthropathies [1, 3]. The persistence of arthralgia for more than 3 months indicates the transition to the chronic stage of chikungunya. In addition to joint pain, exacerbation of comorbidities, tenosynovitis, tendinitis, and neuritis have been reported in patients in the chronic phase of the disease [2–4]. Before the epidemic in the Indian Ocean islands (the Comoros, Mauritius, the Seychelles, Madagascar, Mayotte, and Reunion) and in India between 2005 and 2006, there were no consistent reports of severe cases or chikungunya-related deaths [5, 6]. Particularly, the chikungunya outbreak in Reunion Island revealed unknown characteristics of the disease, such as arthralgia persisting for more than 15 months (with critical implications in quality of life) and high lethality among elderly patients with preexisting conditions, such as hypertension and diabetes [5, 7]. Corroborating these findings, more than 4,500 excess deaths were estimated to have occurred in Ahmedabad, India [8], and Mauritius [9] during the 2005–2006 chikungunya epidemics.

These findings were published at least 4 years before the virus hit the island of Saint Martin in September 2013. Yet the American and Caribbean preparedness and response to the introduction of CHIKV was precarious; as of 2017, only Canada, Cuba, and Chile did not observe autochthonous cases in the region, and the Pan American Health Organization (PAHO) reported 631 chikungunya deaths among 2.5 million cases [10]. This number of deaths, however, is largely underestimated. Considering only 3 states of northeastern Brazil (Pernambuco, Rio Grande do Norte e Bahia) and 4 countries/territories in the Americas (Dominican Republic, Puerto Rico, Guadeloupe, and Martinique), an excess mortality of more than 14,000 deaths during chikungunya epidemics has been estimated for the years 2014 to 2016, mainly concentrated among the elderly [11–14]. There is no comprehensive estimate of excess mortality for Colombia and Brazil (after 2016), the countries that recorded the largest CHIKV epidemics, along with the Dominican Republic. Nonetheless, if a similar excess mortality pattern is considered, more than 35,000 deaths caused primarily or secondarily by CHIKV infection may have occurred in the Americas and the Caribbean in about 4 years (2014–2017).

## Why were chikungunya-related deaths not reported?

Critical reasons for death underreporting include incipient epidemiological surveillance systems, the scarcity of human and laboratory resources, and limited health network infrastructure [12]. In addition, some aspects of the clinical course of chikungunya contribute to death underreporting, such as (i) the fatal association of chikungunya with the decompensation of comorbidities leads to death certificates that only list prior conditions or the immediate cause of death; (ii) severe cases that require long-term hospitalization may result in a fatal nosocomial infection, which is then reported as the cause of death; and (iii) children without atypical clinical manifestations, especially small infants who cannot adequately express their symptoms, are less likely to have a diagnosis of acute chikungunya. Also, chikungunya-related deaths occur at different stages of the disease (as opposed to dengue, when a death often occurs within the first 10 days [15]), and most of the fatal victims are older than 75 years [7, 13]—elderly deaths are usually considered as inevitable events, do not trigger media attention or widespread commotion, and rarely are followed by a rigorous investigation, contrary to what can happen when victims are young.

## Chikungunya-related deaths in Fortaleza, Brazil

**Fig 1:**
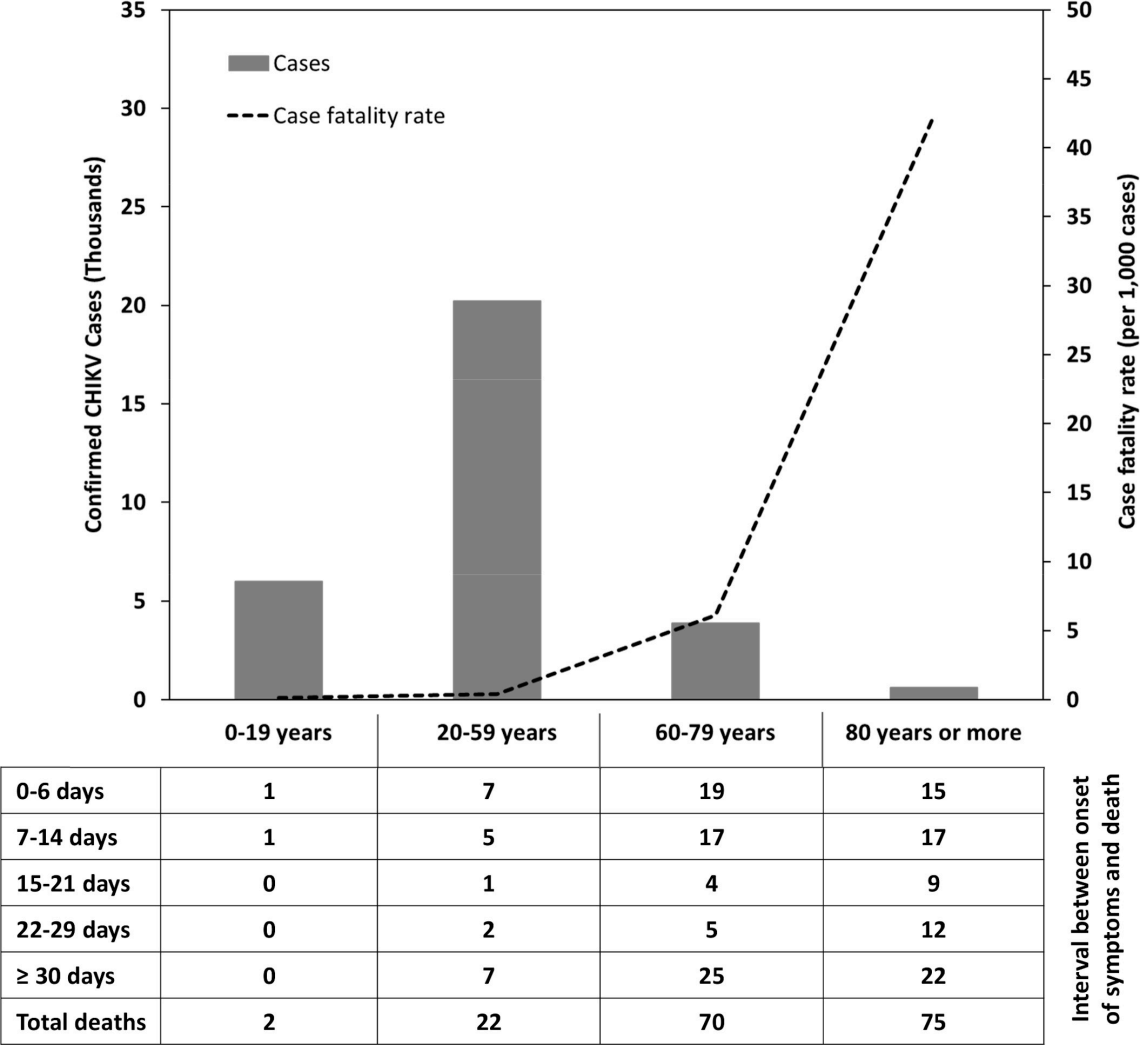
Confirmed chikungunya cases (N = 80,153), case fatality rates (per 1,000 cases), and confirmed chikungunya deaths (N = 169) by age groups and interval between onset of symptoms and death (days) in Fortaleza, Brazil (2016–2017).

The magnitude of problem is revealed by the analysis of data reported by the Department of Epidemiological Surveillance of Fortaleza, the fifth largest city in Brazil (capital of Ceará state) that had a chikungunya epidemic in 2016–2017 (80,000 cases), recording the highest number of chikungunya-related deaths in the Americas (169 deaths), with a median age of death of 78 years. The Epidemiological Bulletin released by the Fortaleza Health Secretariat (https://saude.fortaleza.ce.gov.br/infosaude-menu/81-boletim-de-saude-de-fortaleza) with accumulated cases from 2016 until epidemiological week 52 of 2017 (Fig 1) reported a case fatality rate (CFR) of 0.6% among those between 60–79 years (70/11,457), and approximately 4% for those older than 80 (75/1,771), similar to the CFR estimated by one of the studies that investigated excess mortality during chikungunya epidemics [12]. It also reported a median time between onset of symptoms and death of 15 days, with a little more than half of the deaths occurring in the acute stage (Fig 1). This wide time interval suggests that unfavorable outcomes may be related to different pathophysiological processes. On the one hand, chikungunya deaths caused primarily by acute CHIKV infection with direct impairment of organs (hepatitis, meningoencephalitis, myocarditis/pericarditis, nephritis) are likely to occur in the acute phase, in a similar mechanism to what has been observed in some fatal cases of dengue [5, 15, 16]. On the other hand, deaths following longer clinical courses (post-acute and chronic stages) would be associated with gradual decompensation of comorbidities and long-term hospitalizations that could eventually culminate in nosocomial infections [5, 16].

## Why was Fortaleza different?

Fortaleza was not an outlier but a unique case of a proper surveillance response to emerging threats. In 2008, a Death Investigation Committee (including infectious disease specialists, virologists, and pathologists) was created by the state to review all dengue-suspected deaths, later expanded to include other arboviruses-suspected deaths. Therefore, all reported chikungunya-related deaths in Fortaleza were thoroughly analyzed by the committee; about 85% of the deaths have met case definition (acute fever and intense arthralgia) with at least one positive laboratory test for recent infection in vivo (reverse transcription-polymerase chain reaction [RT-PCR] and/or immunoglobulin M [IgM]) and/or postmortem confirmation (immunohistochemistry); the other 15% had clinical records and data from epidemiological investigation. In addition, the committee described the clinical course of patients in a timeline format between the onset of chikungunya symptoms and the death in order to try to establish an association between CHIKV infection and the recorded cause of death of each patient. The committee’s work supported by postmortem diagnosis substantially reduced the underreporting of deaths due to dengue, Zika virus, and chikungunya in Fortaleza [17]. Unfortunately, very few municipalities/localities in Brazil or in any other country have undertaken similar efforts to elucidate arboviruses deaths.

## What should be done to prevent chikungunya fatality?

Despite the need for further studies to establish a definite causal link between CHIKV and mortality, current evidence suggest that chikungunya may be the vector-borne disease associated with the highest number of deaths in the shortest time in the Americas. Nevertheless, until now, neither the World Health Organization nor PAHO have issued an alert for the lethality of the disease. Currently, most clinical protocols to manage chikungunya cases are focused on acute or chronic joint pain relief. Treatment of severe or atypical cases mainly recommend hospitalization in intensive care units for symptomatic treatment and compensation of altered vital functions. While relieving painful symptoms is critical to the quality of life of most patients, intermittent symptomatic treatment, without adequate individual follow-up, does not appear to have an effect on mortality reduction. Moreover, deaths can happen in very few days following onset of symptoms.

It is imperative that international agencies formally recognize the high lethality of chikungunya in specific groups in order to reorient disease surveillance. Although usually a benign disease, chikungunya does not always have a self-limited clinical course. CHIKV infection and its complications can be the underlying cause of death or trigger a decompensation of preexisting medical conditions leading to a fatal outcome. Current chikungunya clinical protocols should be reviewed with the ultimate aim to prevent deaths [12, 16]. So far, France and Brazil have issued revised guidelines to manage chikungunya, following epidemics in the French South American and Pacific territories and in Brazil [3, 18]. However, contrary to dengue protocols, chikungunya management guidelines are not yet targeted to avoid fatal outcomes.

Considering the complexity of the clinical course of chikungunya, and in the absence of antivirals, efforts should be undertaken to make continuous treatment, specific for each stage of the disease (acute, post-acute, and chronic), in order to prevent deaths. Protocols must be regularly updated as new knowledge is produced so that treatment follows the most current evidence. In addition, it is important to create a patient mortality risk classification based on the available knowledge. While we acknowledge that scant histopathological data and knowledge gaps in the pathophysiology of chikungunya limit the comprehensiveness of the risk classification, available knowledge already offers a unique opportunity to mitigate mortality, particularly among elderly patients with specific comorbidities.

Currently, CHIKV transmission in the Americas is very low. Yet it is expected that new outbreaks will emerge as the virus spreads to new areas (in the Americas and other regions) and as the susceptible population in previously affected areas builds up. In anticipation of new epidemics, it is critical that new protocols of chikungunya management be devised, with a particular attention to mortality prevention, especially among well-known high-risk groups. Failure to do so will result, once again, in an unacceptable number of chikungunya-related deaths. While most of these deaths may not get reported, as recently happened, all will be foretold. Time will tell.
